# Oral sucrose and a pacifier for pain relief during simple procedures in preterm infants: a randomized controlled trial

**DOI:** 10.4103/0256-4947.52821

**Published:** 2009

**Authors:** Fathia A. Elserafy, Saad A. Alsaedi, Julita Louwrens, Bakr Bin Sadiq, Ali Y. Mersal

**Affiliations:** a From the Clinical Pharmacy Department, School of Pharmacy, King Abdulaziz University, Jeddah, Saudi Arabia; b From the Department of Pediatrics, King Abdulaziz University Hospital, Jeddah, Saudi Arabia; c From the Neonate Intensive Care Unit, Jeddah, Saudi Arabia; d From the Research Centre, Jeddah, Saudi Arabia; e From the Department of Pediatrics, King Faisal Specialist Hospital and Research Centre-Jeddah, Jeddah, Saudi Arabia

## Abstract

**BACKGROUND AND OBJECTIVES:**

Previous randomized trials of the analgesic effects of sucrose, glucose, and a pacifier in term neonates have shown that the pacifier resulted in lower pain scores than glucose or sucrose, but the pacifier with and without sucrose did not differ. The current study was designed to assess the analgesic effect of pharmacologic (sucrose, water) and a non-pharmacologic measures (pacifier) in preterm infants and to find whether there is any synergism between these intervention in relieving pain during painful procedures.

**PATIENTS AND METHODS:**

In this double-blind, randomized, controlled study, 36 preterm infants (mean 31 weeks gestational age, range 27 to 36 weeks) were randomly allocated to six different regimens (0.5 mL sterile water with pacifier, 0.5 mL sterile water without pacifier, 0.5 mL sucrose 24% with pacifier, 0.5 mL sucrose 24% without pacifier, pacifier alone and control group) during a stay in intensive care of up to 15 days. Pain scores were measured with the Premature Infant Pain Profile (PIPP), a validated behavioral acute pain scale.

**RESULTS:**

Of all the regimens, the lowest pain scores occurred with the use of 24% sucrose solution combined with pacifier. The mean pain score for the combination of sucrose with pacifier was 0.7 as compared to 1.4 for the sterile water with pacifier group (*P*<.05).

**CONCLUSION:**

The synergistic effect of the combination of sucrose and non-nutritive sucking was clinically effective and safe in relieving the pain of simple procedures such as venipuncture or heel stick in preterm and term infants, but further research is needed on these interventions alone and in combination with other behavioral interventions in neonates.

Treating pain in the newborn is essential for many reasons, both clinical and ethical. Pain can lead to decreased oxygenation, hemodynamic instability, and increased intracranial pressure.^1^ For neonates receiving intensive care, it is widely accepted that central analgesics, administered intravenously, should be used to relieve pain.^1^ However, infants who are less sick or who are not in neonatal intensive care units usually do not receive an analgesic for painful procedures. Obviously, central analgesics cannot be used for pain associated with occasional blood sampling performed in newborns not in intensive care. It is therefore essential to find simple, acceptable, and well-tolerated methods to reduce pain in these infants.^1^ Non-nutritive sucking during heel prick procedures decreases behavioral distress in the newborn infant.^2^ Studies support the theory that sucrose (due to the sweet taste) and pain relief are interrelated through the body's endogenous opioid system which provides natural analgesia.^3^–^9^ The analgesic effect of sucrose is reversed with administration of naloxone, an opioid antagonist, suggesting that sucrose activates the central endogenous opioid system, with an action similar to that of opioid analgesics.^10^,^11^ Our objective was to assess and compare the analgesic effects of sucrose versus sterile water alone or with a pacifier in relieving pain in preterm infants prior to painful procedures. We also sought to determine whether there is a synergistic effect with oral sucrose when combined with non-pharmacologic interventions to relieve pain during painful procedures in preterm infants.

## PATIENTS AND METHODS

This was a randomized, prospective, double-blinded, controlled study of the analgesic effects of sucrose versus sterile water alone or with a pacifier in preterm infants admitted to the neonatal intensive care unit (NICU) of a tertiary care hospital. The study protocol and consent form were approved by the hospital institutional review board. Recruitment was done between January 2005 and May 2007. The inclusion criteria were that subjects had to be preterm infants of less than 37 weeks of gestational age admitted to the NICU at King Faisal Specialist Hospital, Jeddah. There had to be a signed parental consent prior to enrollment. Exclusion criteria were: 1) exposure antenatally to maternal sedation (opioid); 2) occurrence of any procedure performed within 24 hours in preterm infants whose mothers had had general anesthesia during delivery; 3) the presence of major neurologic abnormalities; 4) Apgar scores at 5 minutes of <5; 5) presence of necrotizing intestinal colitis; 6) nothing by mouth status for any reason and 7) being preterm with hyperglycemia.

Of 48 preterm infants of <37 weeks gestational age admitted and expected to be included in this study, 36 infants (72%) completed the study. The other 12 infants were eligible but they did not complete the study because of failure to get parental consent. Every patient participating in the study received each of six different regimens during a maximum stay of 15 days from admission or the end of the NICU stay (which-ever came first). The solutions were prepared and coded in the pharmacy and none of the investigators or family of the patient knew the identity of the solution. Documentation of the identity of the solution was kept in a closed cabinet that was opened only at the time of analyzing the results. The nurse opened a consecutively numbered envelope (all envelopes were previously prepared with codes for the six treatment regimens, each in a folded paper). For each assignment, a paper was randomly picked so that assignments were random and doublebblinded for the sucrose and water solutions. The six regimens were 0.5 mL sterile water with pacifier, 0.5 mL sterile water without pacifier, 0.5 mL sucrose 24% with pacifier, 0.5 mL sucrose 24% without pacifier, pacifier alone (standard nipple stuffed with gauze square for resistance, held in the infant's mouth for 2 minutes prior the procedure and kept gently in the infant's mouth throughout the procedure), and standard of care or no treatment (control group). For the sucrose and water solutions, the tip of a 1 mL syringe without the needle was placed in the infant's mouth and the solution was instilled with gentle movements of the syringe to stimulate sucking for 30 seconds. Each treatment was given 2 minutes prior to the procedure.

**Figure 1:**
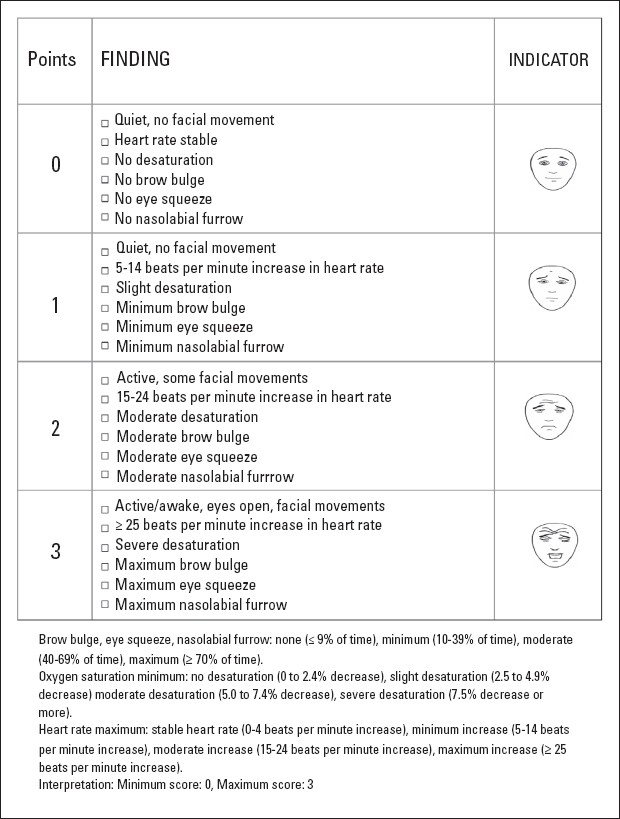
The Premature Infant Pain Profile (PIP) pain evaluation tool.^12^

The primary outcome measure was the evaluation of pain induced by venipuncture using the Premature Infant Pain Profile (PIPP). Using this scale, the neonate is observed for 15 seconds and the following seven indicators are scored: gestational age, behavioral state, heart rate increase from baseline, oxygen saturation change from baseline and duration of time that the infants have brow bulge, eye squeeze and a naso-labial furrow (Figure 1).^12^ Heart rate was measured by pulse oximetry, and considered only when the quality of the registered wave was adequate. Bradycardia was defined by a heart rate less than 80 beats per minute and tachycardia by a heart rate above 160 beats per minute. Oxygen saturation was evaluated by pulse oximetry, taking into account the quality of wave registered. Hypoxia was defined by an oxygen saturation below 88% (an oxygen saturation 15% less than the patient's baseline was used in patients with cyanotic heart disease). The physiologic and behavioral pain parameters were evaluated at six different times: immediately prior the procedure, during venipuncture, one minute after the procedure, three minutes after the procedure, five minutes after the procedure, and ten minutes after the procedure. The response time (crying time) was assessed at 0, 1, 3, 5, and 10 minutes.

Repeated measures analysis of variance was used for the statistical analysis. Pain score comparisons between different treatments were summarized using mixed between-within ANOVA. Results were significant when *P<.05.* Because the F statistic is based on the ratio of betweenbgroup variance to within-group variance, there must be a very large between-group difference to attain a significant result; that is, the large variability among the subjects could obscure any real differences between the groups. This is especially true if the groups are small. One way to remove these individual differences is to assign each subject to all treatments with each subject exposed to medications 1 through 5 and the control in random order. Each subject serves as their own control, and the within or error variance is decreased. This results in a more powerful test and decreases the number of subjects needed for the study.^13^ The pre-procedure pain score means ranged from 0.037 to 0.15 in all groups so these scores were not included in further analysis due to the small values. Further analysis was done for the five treatment times (0, 1, 3, 5, and 10 minutes).

## RESULTS

**Figure 2:**
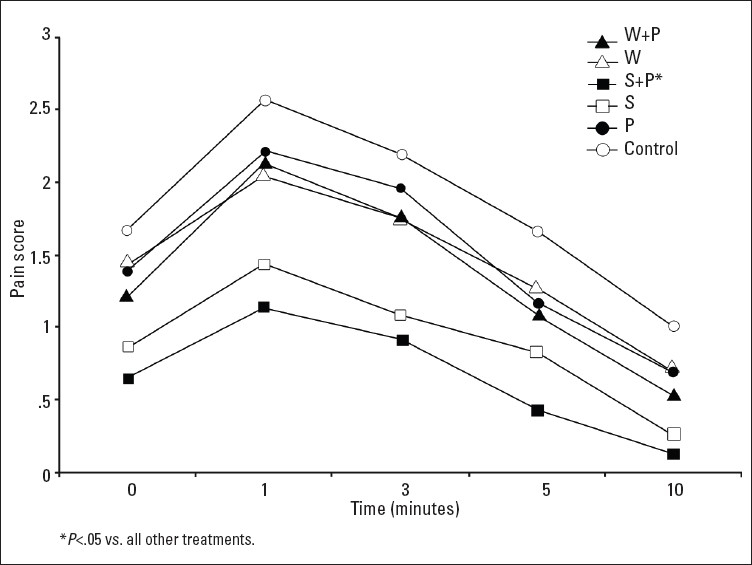
Pain scores for treatment regimens (P: pacifier, S: sucrose 24%, W: sterile water).

Demographic and clinical characteristics of the 36 preterm infants are shown in Table 1. There were no significant differences between treatment groups in heart rate, blood pressure, O_2_ saturation and glucose measurement (*P*>.05) (Table 2), but there were significant differences in crying time and pain score (*P*=.001). Pain scores were hghest at 1 minute past painful stimuli for all five treatment groups (Figure 2). The use of 24% sucrose solution combined with pacifier resulted in the lowest pain score of all groups for all measurements from zero to ten minutes (*P*<.05). In all treatment groups, the lowest pain-scores were pretreatment scores with the means ranging from 0.037 to 0.15. Pain scores elevated gradually at zerobtime (range, 0.69 to 1.69) and elevated even further at 1 minute to reach a peak (range, 1.077 to 2.52). At 3 minutes they dropped (range, 0.85 to 2.1) and dropped further at 5 minutes (range, 0.38 to 1.5) There was a change in pain scores over the five time periods (main effect for time) and this change was statistically significant (Wilks lambda=0.145, F=214.6, *P*<.0005, multivariate partial eta squared =0.855). There was also a statistically significant difference in pain scores between treatment groups [F (5, 149)=14.75, *P*=.0005] and the change in pain scores over time was different for the treatment groups with an interaction effect (Wilks lambda=0.63, F=3.56, *P*<.0005).

**Table 1:** Demographic and clinical characteristics of 36 preterm babies.

	Median (range)	Mean (SD)
Apgar score	8.5 (8-10)	8.6 (0.6)
Body weight (kg)	1.7 (0.6-2.5)	1.7 (0.6)
Gestational age (weeks)	32 (27-36)	32.4 (2.9)

**Table 2:** The mean values for Premature Infant Pain Profile measurements during the six different treatments.

	Sterile water + pacifier	Sterile water	Sucrose 24% + pacifier	Sucrose 24%	Pacifier alone	Control	*P*
Heart rate (beats/min)	160.5	161.8	155.3	156.9	162.2	163.1	.167
O_2_ saturation (%)	90.9	91.3	91.4	91.2	90.6	91.5	.979
Respiratory rate (breaths/min)	50.8	54.4	48.2	46.3	48.8	50.3	.193
Crying time (s)	11.3	11.5	4.6	6.1	11.8	17.4	.001
Blood pressure(mm Hg)	50.2	48.9	45.4	48	46.9	46.7	.246
Glucocheck (mmol/L)	4.5	4.8	5.5	5.1	4.7	4.9	.227

Values are means.

## DISCUSSION

In ths study, oral sucrose administration before an acute painful procedure decreased the behavioral pain indicators and composite pain scores in neonates undergoing heel stick and/or venipuncture as measured by the PIPP. The pain score was significantly reduced in infants who received 0.5 mL (0.12 gm) of 24% sucrose solution with mean pain score of 0.9 at the 0.05 level of significance. The combination of pacifier and sucrose 24% solution showed more clinical effect and reduced the mean crying time to 4.6 seconds (Table 2) which was statistically significant (*P*=.001).

Similar results were described in previous studies where sucrose was found to be safe and effective in newborn infants for reducing procedural pain from single painful events such as heel lance or venipuncture.^14^,^15^ Sucrose provided an additional benefit when used with a pacifier in infants aged 1-3 months undergoing venipuncture in a pediatric emergency department.^16^ A modest 16% reduction in overall pain among newborns of both diabetic and nondiabetic mothers was reported when sucrose was used repeatedly for all procedures performed in the first two days after birth, but when each procedure was evaluated separately, the effectiveness of sucrose was limited to venipuncture.^17^

The use of sterile water alone or pacifier alone or the combination of sterile water and pacifier during painful stimuli were not statistically different in their effect on pain relief and were not better than the control group that received the standard of care or no pain control for simple procedures. The mean pain score was 1.4, 1.4, 1.5 and 1.8 respectively. However, one review recently concluded that the overall effects of non-nutritive sucking on pain related to minor procedures were generally superior to standard care.^18^ Reports included in this review found that relative to standard care, non-nutritive sucking resulted in reduced crying behavior, reduced heart rate and lower behavioral distress. However, several methodological problems were also noted in this review as the authors of these reports evaluated crying behaviors using a subjective, unvalidated scale.^2^,^19^,^20^

Arranging all the treatment groups in our study with respect to the mean pain score showed that the highest pain score occurred at one minute post-painful stimuli with all the treatment groups, but the maximum pain occured with the control group or with the use of the pacifier alone for pain relief with a pain score of 2.5 and 2.2, respectively. The lowest pain score occurred at 10 minutes with a mean pain score of 0.1, 0.3, 0.5, 0.7, 0.7 and 1 with the use of sucrose 24% combined with pacifier, sucrose 24% solution, sterile water combined with pacifier, sterile water alone, pacifier alone and control group respectively. In preterm infants, it has also been reported that the provision of sucrose and a pacifier is more effective than sucrose alone in reducing procedural pain as measured by the PIPP score in three reports.^9^,^21^,^22^

It is important to emphasize that using 24% sucrose has no side effects and is safe for preterm analgesia. This is supported by evidence reported in the literature where sucrose was a useful and safe analgesic for minor procedures in neonates given 2 mL of 25% sucrose solution before venipuncture, which significantly reduced crying time.^23^ Currently the standard practice during venipuncture in newborn infants is not to use any analgesia. This study demonstrated the ease of use of sucrose and/or pacifier use, which should inspire a change in practice toward the use of routine sucrose analgesia during the first weeks of life in preterm infants. This practice is supported by published guidelines for pain relief using sucrose analgesia during minor procedures such as heel stick or venipuncture. These guidelines state that dipping the pacifier in 24% sucrose solution or using oral syringe application directly on the tongue, each dip >0.2 mL applied 1 to 2 minutes before or during painful procedure. Sucrose administration can be repeated as needed for pain relief.^24^

The synergistic effect of the combination of sucrose and non-nutritive sucking is a statistically and clinically effective and safe intervention for relieving pain during simple procedures as venipuncture or heel stick in preterm and term infants. Additional research is needed on the efficacy and safety of implementing these interventions, alone and in combination with other behavioral interventions (e.g. facilitated tucking, kangaroo care) in neonates. In addition, research is needed on the influence of implementing these interventions on pain response and clinical outcomes in very low birth weight neonates in the NICU.

## References

[1] Carbajal R, Chauvet X, Couderc S, Olivier-Martin M (1999). Randomized trial of analgesic effects of succrose, glucose and pacifiers in term neonates. BMJ.

[2] Corbo G, Mansi G, Stagni A, Romano A, van den Heuvel J, Capasso L, Raffio T, Zoccali S, Paludetto R (2000). Non-nutritive sucking during heel stick procedures decreases behavioral distress in the newborn infant. Biol Neonate.

[3] Barr R, Pantel M, Young S, Wright JH, Henddricks LA, Gravel R (1999). The response of crying newborns to sucrose: Is it a “sweetness” effect?. Physiol Behav.

[4] Nikfar S, Abdollahi M, Etemad F, Sharifzadeh M (1997). Effects of sweetening agents on morphine-induced analgesia in mice by formalin test. Gen Pharmacol.

[5] Bucher H, Moster T, Siebenthal K, Keel M, Wolf M, Duc G (1995). Sucrose reduces pain reaction to heel lancing in preterm infants: a placebo-controlled, randomized and masked study. Pediatr Res.

[6] Abad F, Diaz N, Domenech E, Robayna M, Rico J (1996). Oral sweet solution reduces pain-related behavior in preterm infants. Acta Paediatrica.

[7] Ramenghi L, Wood C, Griffith G, Levene MI (1996). Reduction of pain response in premature infants using intraoral sucrose. Arch Dis Childhood.

[8] Ramenghi L, Evans D, Levene M (1999). Sucrose analgesia: absorptive mechanism or taste percception?. Arch Dis Childhood Fetal Neonatal Ed.

[9] Stevens B, Johnston C, Franck L, Petryshen P, Jack A, Foster G (1999). The efficacy of developmentally sensitive interventions and sucrose for relieving procedural pain in very low birth weight neonates. Nurs Res.

[10] Barr R, Young S, Wright J, Cassidy KL, Henddricks L, Bedard Y, Yaremko J, Leduc D, Treherne S (1995). Sucrose analgesia and diphtheria-tetanus-pertussis immunizations at 2 and 4 months. J Dev Behav Pediatr.

[11] Blass E, Shah A (1995). Pain-reducing properties of sucrose in human newborns. Chem Sen.

[12] Stevens B, Johnston C, Petryshen P, Taddio A (1996). Premature infant pain profile: development and initial validation. Clin J Pain.

[13] Munro BH (2005). Statistical Methods for Health care Research.

[14] Stevens B, Yamada J, Ohlsson A (2004). Sucrose for analgesia in newborn infants undergoing painful procedures [CD-RO M]. Cochrane Data-base Syst Rev.

[15] Acharya A, Annamali S, Taub N, Field D (2004). Oral sucrose analgesia for preterm infant venepunctture. Arch Dis Child Fetal Neonatal Ed.

[16] Curtis S, Jou H, Ali S (2007). A randomized controlled trial of sucrose and/or pacifier as analgesia for infants receiving venipuncture in a pediatric emergency department. BMC Pediatr.

[17] Taddio A, Shah V, Hancock R, Smith RW, Stepphens D, Atenafu E, Beyene J, Koren G, Stevens B, Katz J (2008). Effectiveness of sucrose analgesia in newborns undergoing painful procedures. CMAJ.

[18] Pinelli J, Symington A, Ciliska D (2002). Non-nutritive sucking in high-risk infants: benign intervention or legitimate therapy?. J Obstet Gynecol Neonatal Nurs.

[19] Miller D, Anderson C (1993). Non-nutritive sucking: effects on crying and heart rate in incubated infants requiring assisted mechanical ventilation. Nurs Res.

[20] Field T, Goldson E (1984). Pacifying effects of non-nutritive sucking on term and preterm neonates during heelstick procedures. Pediatr.

[21] Gibbins S, Stevens B, Hodnett E, Pinelli J, Ohlsson A, Darlington G (2002). Efficacy and safety of sucrose for procedural pain relief in preterm and term neonates. Nurs Res.

[22] Stevens B, Yamada J, Beyene J (2005). Consistent management of repeated procedural pain with sucrose in preterm neonates: is it effective and safe for repeated use over time?. Clin J Pain.

[23] Taksande A, Vilhekar K, Jain M Sucrose as an analgesics in newborn infants.

[24] Lefrak L, Burch K, Caravantes R, Knoerlein K, DeNolf N, Duncan J, Hampton F, Johnston C, Lockey D, Martin-Walters C, McLendon D, Portter M, Richardson C, Robinson C, Toczylowski K (2006). Sucrose analgesia: identifying potentially better practices. Pediatr.

